# Supplementary material for: Genomic signatures of domestication in Old World camels
Source: Commun Biol. 2020 Jun 19;3:316. doi: 10.1038/s42003-020-1039-5 (PMC7305198; doi:10.1038/s42003-020-1039-5)
Supplement: Supplementary file 1 — Supplementary Information [file 42003_2020_1039_MOESM1_ESM.pdf]

Supplementary Figures

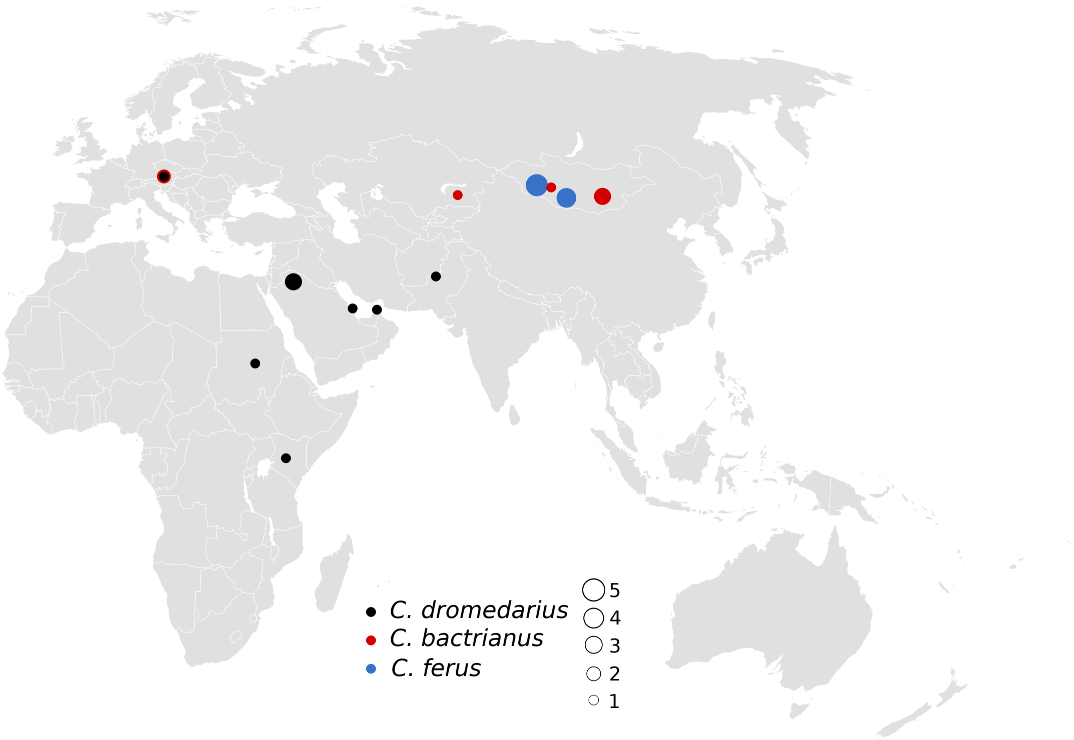

Supplementary Figure 1. Map illustrating the samples used in this study for genome sequencing. The size of each circle is proportional to the sample size for that location. Colors correspond to the three Old World camel species.

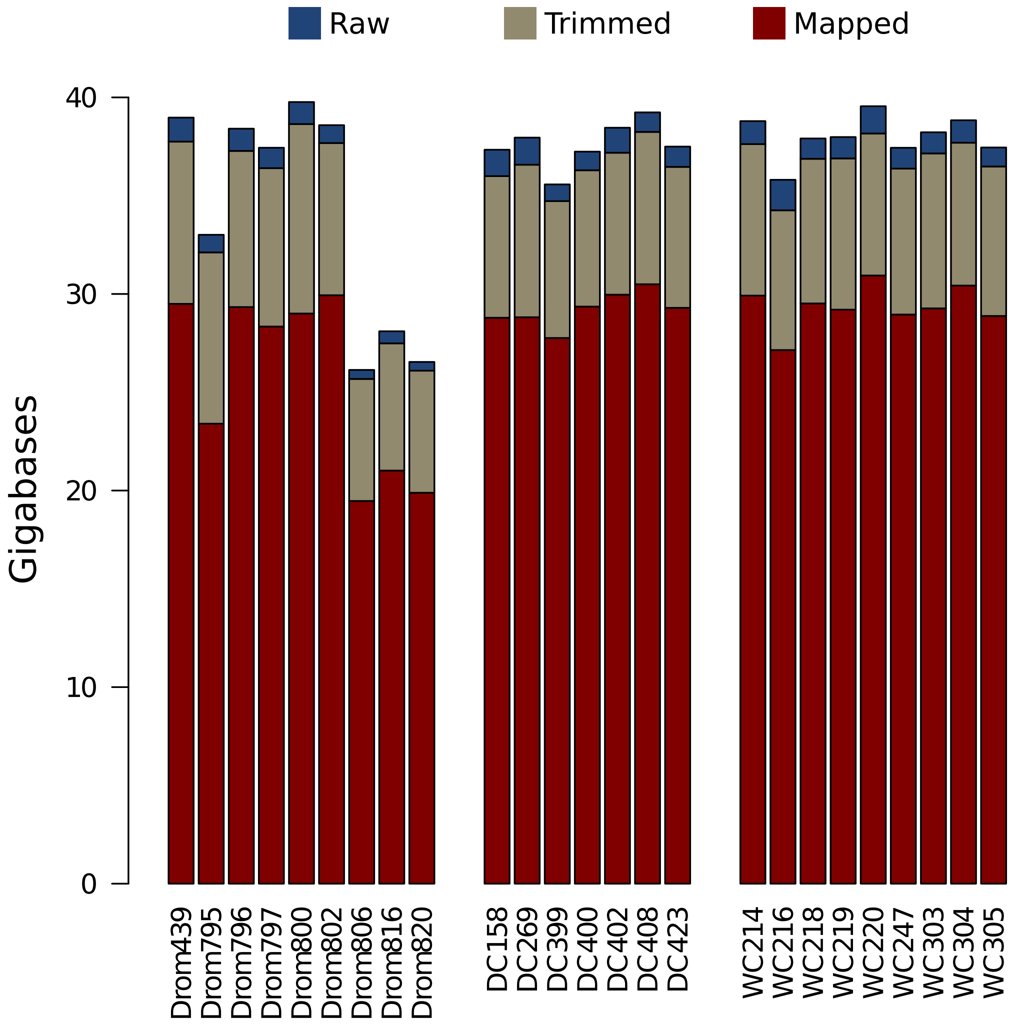

Supplementary Figure 2. The number of raw, trimmed, and mapped bases for each individual. See Supplementary Table 1 for the list of sequenced camels.

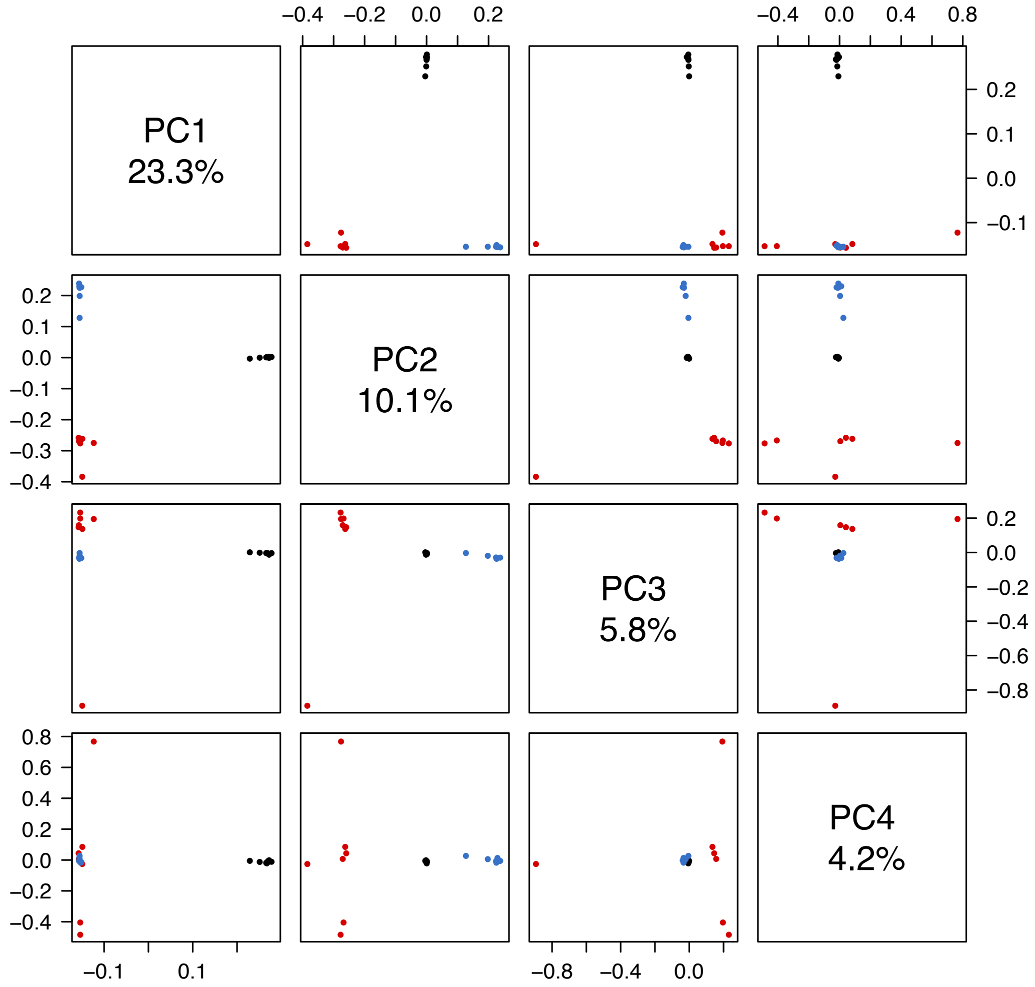

Supplementary Figure 3. Principal components analysis of genetic variation in Old World camels. The first four principal components (PC) are shown as pairwise plots, with the diagonal stating the variation explained by the PC. Dromedaries (*Camelus dromedarius*) are shown as black, domestic Bactrian camels (*Camelus bactrianus*) as red, and wild camels (*Camelus ferus*) as blue circles.

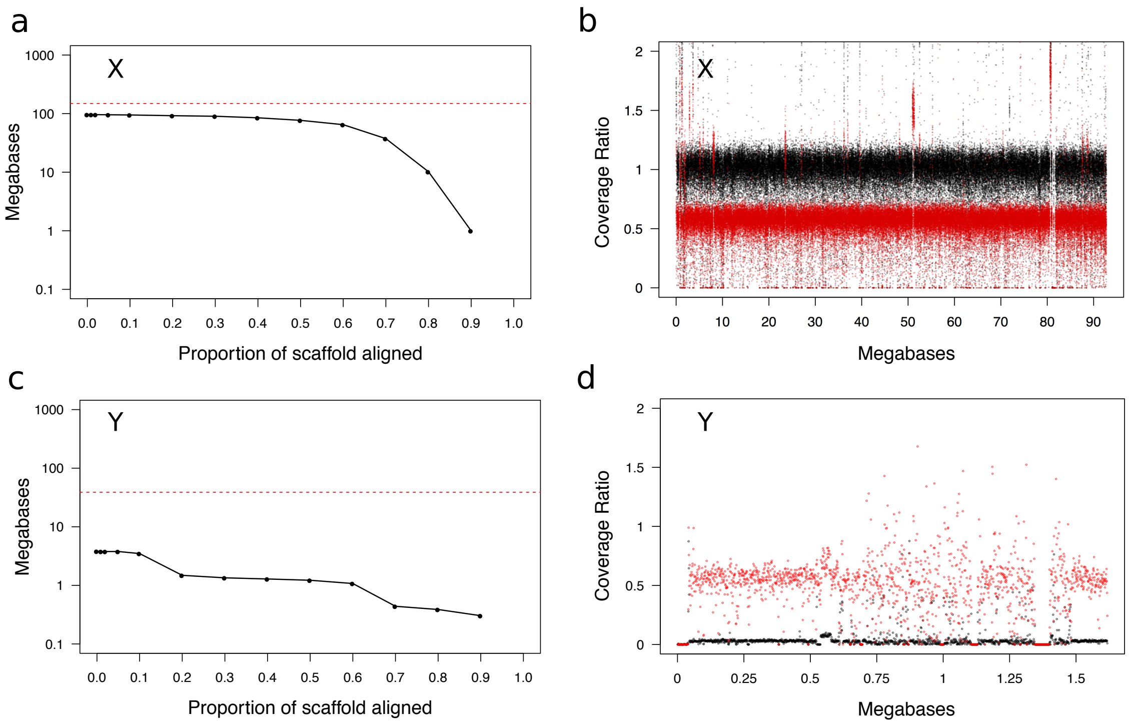

Supplementary Figure 4. Identification of X and Y putative scaffolds. (a,b) Total number of megabases contained in scaffolds that aligned to the cow (*Bos taurus*) reference X (a) and Y (b) chromosome sequences. (c,d) The ratio of sequencing coverage in putative X (c) and Y (d) scaffolds relative to the genome-wide background in female (black) and male (red) individuals. Notice male coverage is ~0.5 the genome-wide background for the putative X scaffolds, and approximately 0 and 0.5 the genome-wide background for the putative Y scaffolds in females and males, respectively.
