# Supplementary material for: Genomic signatures of domestication in Old World camels
Source: Commun Biol. 2020 Jun 19;3:316. doi: 10.1038/s42003-020-1039-5 (PMC7305198; doi:10.1038/s42003-020-1039-5)
Supplement: Supplementary file 12 — Description of Additional Supplementary Files [file 42003_2020_1039_MOESM12_ESM.pdf]

Description of Additional Supplementary Files

Supplementary Data 1. Description of the 25 Old World camel samples used in this study.

Supplementary Data 2. Summary of the raw and cleaned sequencing data, and the mapping results for each camel individual. MQ = Phred-scaled mapping quality score.

Supplementary Data 3. Summary of the positively selected genes identified in dromedaries using a combination of the homogeneity and HKA tests. Transcript = GenBank accession number; A = number of polymorphic sites in the dromedary samples; B = number of polymorphic sites in the wild camel samples; C = number of fixed differences between dromedaries and both wild camels and the alpaca genome sequence; D = number of fixed differences between wild camels and both dromedaries and the alpaca genome sequence; Homo_P = p-value from the Fisher exact test for 2x2 contingency table; HKA_Drom_P = p-value from the HKA test within the dromedaries; HKA_WC_P = p-value from the HKA test within the wild camel; OMIA = domestic species disease or disorder [accession ID number] associated with this gene according to the Online Mendelian Inheritance in Animals database (https://www.omia.org); OMIM = Name [accession ID number] of diseases or disorders associated with this gene according to the Online Mendelian Inheritance in Man database (https://www.omim.org); GO_Terms = Gene Ontology (GO) terms annotated to the gene using Blast2GO; Entrez = gene description from the NCBI Entrez database; Uniprot = gene description from the Uniprot database.

Supplementary Data 4. Results of the Gene Ontology (GO) enrichment analysis using TopGO for the positively selected genes from Table S1 in dromedaries. P values (P) were corrected using the false discovery rate (FDR).

Supplementary Data 5. Summary of the positively selected regions identified in dromedaries from a comparison of nucleotide diversity (π; 0.5 percentile) and divergence (Dxy; 99.5 percentile). Scaffold = GenBank accession number of the scaffold from the C. ferus genome; OMIM = Name [accession ID number] of diseases or disorders associated with this gene according to the Online Mendelian Inheritance in Man database (https://www.omim.org); GO_Terms = Gene Ontology (GO) terms annotated to the gene using Blast2GO; Entrez = gene description from the NCBI Entrez database; Uniprot = gene description from the Uniprot database.

Supplementary Data 6. Results of the Gene Ontology (GO) enrichment analysis using TopGO for the positively selected genes from Table S3 in dromedaries. P values (P) were corrected using the false discovery rate (FDR).

Supplementary Data 7. Summary of the positively selected regions identified in domestic Bactrian camels using the population branch statistic. Scaffold = GenBank accession number of the scaffold from the C. ferus genome; OMIM = Name [accession ID number] of diseases or disorders associated with this gene according to the Online Mendelian Inheritance in Man database (https://www.omim.org); GO_Terms = Gene Ontology (GO) terms annotated to the gene using Blast2GO; Entrez = gene description from the NCBI Entrez database; Uniprot = gene description from the Uniprot database.

Supplementary Data 8. Results of the Gene Ontology (GO) enrichment analysis using TopGO for the positively selected genes from Table S5 in domestic Bactrian camels. P values (P) were corrected using the false discovery rate (FDR).

Supplementary Data 9. Summary of the positively selected regions identified in wild camels. Scaffold = GenBank accession number of the scaffold from the C. ferus genome; OMIM = Name [accession ID number] of diseases or disorders associated with this gene according to the Online Mendelian Inheritance in Man database (https://www.omim.org); GO_Terms = Gene Ontology (GO) terms annotated to the gene using Blast2GO; Entrez = gene description from the NCBI Entrez database; Uniprot = gene description from the Uniprot database.

Supplementary Data 10. Complete list of the 17,912 canonical (longest isoforms) genes annotated in C. ferus and used in this study and their associated annotations. Transcript = GenBank accession number; GO_Terms = Gene Ontology (GO) terms annotated to the gene using Blast2GO.
