# Supplementary material for: Genomic signatures of domestication in Old World camels
Source: Commun Biol. 2020 Jun 19;3:316. doi: 10.1038/s42003-020-1039-5 (PMC7305198; doi:10.1038/s42003-020-1039-5)
Supplement: Supplementary file 14 — Reporting Summary [file 42003_2020_1039_MOESM14_ESM.pdf]

nature research

Corresponding author(s): Robert Fitak, Pamela Burger

Last updated by author(s): May 18, 2020

Reporting Summary

Nature Research wishes to improve the reproducibility of the work that we publish. This form provides structure for consistency and transparency in reporting. For further information on Nature Research policies, see Authors & Referees and the Editorial Policy Checklist.

Statistics

For all statistical analyses, confirm that the following items are present in the figure legend, table legend, main text, or Methods section.

n/a	Confirmed	
☐	☒	The exact sample size (*n*) for each experimental group/condition, given as a discrete number and unit of measurement
☐	☒	A statement on whether measurements were taken from distinct samples or whether the same sample was measured repeatedly
☐	☒	The statistical test(s) used AND whether they are one- or two-sided<br>*Only common tests should be described solely by name; describe more complex techniques in the Methods section.*
☒	☐	A description of all covariates tested
☐	☒	A description of any assumptions or corrections, such as tests of normality and adjustment for multiple comparisons
☐	☒	A full description of the statistical parameters including central tendency (e.g. means) or other basic estimates (e.g. regression coefficient) AND variation (e.g. standard deviation) or associated estimates of uncertainty (e.g. confidence intervals)
☐	☒	For null hypothesis testing, the test statistic (e.g. *F*, *t*, *r*) with confidence intervals, effect sizes, degrees of freedom and *P* value noted<br>*Give P values as exact values whenever suitable.*
☒	☐	For Bayesian analysis, information on the choice of priors and Markov chain Monte Carlo settings
☒	☐	For hierarchical and complex designs, identification of the appropriate level for tests and full reporting of outcomes
☒	☐	Estimates of effect sizes (e.g. Cohen's *d*, Pearson's *r*), indicating how they were calculated

Our web collection on statistics for biologists contains articles on many of the points above.

Software and code

Policy information about availability of computer code

Data collection	No commercial, open-source, or custom code was used to collect or generate data for this study.
Data analysis	A large number of open-source software and custom computer code was used to analyze the data in this study. A full, detailed, and annotated repository of all computer code, including specific versions of open-source software used, is publicly available in GitHub at https://github.com/rfitak/Camel_Genomics .

For manuscripts utilizing custom algorithms or software that are central to the research but not yet described in published literature, software must be made available to editors/reviewers. We strongly encourage code deposition in a community repository (e.g. GitHub). See the Nature Research guidelines for submitting code & software for further information.

Data

Policy information about availability of data

All manuscripts must include a data availability statement. This statement should provide the following information, where applicable:

- Accession codes, unique identifiers, or web links for publicly available datasets
- A list of figures that have associated raw data
- A description of any restrictions on data availability

Raw sequencing data for the 25 genomes in the study are available in GenBank (BioProject accession PRJNA276064). Computer code and scripts for the various analyses are available at GitHub (https://github.com/rfitak/Camel_Genomics). Additional data files generated during the analyses are available in Dryad (https://doi.org/10.5061/dryad.prr4xgxj2).

Field-specific reporting

Please select the one below that is the best fit for your research. If you are not sure, read the appropriate sections before making your selection.

☒ Life sciences ☐ Behavioural & social sciences ☐ Ecological, evolutionary & environmental sciences

For a reference copy of the document with all sections, see nature.com/documents/nr-reporting-summary-flat.pdf

Life sciences study design

All studies must disclose on these points even when the disclosure is negative.

Sample size	A total of 9, 9, and 7 samples were collected for Camelus dromedarius, Camelus ferus, and Camelus bactrianus, respectively. Sample size was determined based upon the difficulty in obtaining specimens form the protected wild camel (C. ferus), availability of funding, and ability to obtain estimates of allele frequencies. Samples were collected with an intent to include a geographically broad representation.
Data exclusions	No data were excluded from this study.
Replication	Not applicable
Randomization	Individual samples were allocated to groups based upon species. No randomization was required.
Blinding	No blinding was required.

Reporting for specific materials, systems and methods

We require information from authors about some types of materials, experimental systems and methods used in many studies. Here, indicate whether each material, system or method listed is relevant to your study. If you are not sure if a list item applies to your research, read the appropriate section before selecting a response.

Materials & experimental systems

n/a	Involved in the study	
☒	☐	Antibodies
☒	☐	Eukaryotic cell lines
☒	☐	Palaeontology
☐	☒	Animals and other organisms
☒	☐	Human research participants
☒	☐	Clinical data

Methods

n/a	Involved in the study	
☒	☐	ChIP-seq
☒	☐	Flow cytometry
☒	☐	MRI-based neuroimaging

Animals and other organisms

Policy information about studies involving animals; ARRIVE guidelines recommended for reporting animal research

Laboratory animals	The study did not involve laboratory animals.
Wild animals	Wild Bactrian camel samples were collected commensally during micro-chipping or radio-collaring within the framework of the legal requirements of both Austria and Mongolia. Micro-chipping of wild camels from the breeding center of the Wild Camel Protection Foundation (WCPF) was performed with the request and consent of the WCPF (John Hare, personal communication). Capture and collaring of wild camels within the Great Gobi Strictly Protected Area "A" was conducted within a cooperation agreement between the International Takhi Group and the Mongolian Ministry of Nature, Environment and Tourism signed on 15.02.2001 and renewed on 27.01.2011. The exact procedure of capture and anesthesia has been published and is cited in the manuscript: Walzer, C., Kaczensky, P., Enkhbileg, D. & Yadamsuren, A. in Camels in Asia and North Africa: Interdisciplinary perspectives on their past and present significance (eds E. Knoll & P. A. Burger) 61-68 (Austrian Academy of Science Press, 2012).
Field-collected samples	All blood samples for domesticated camelid species were retrieved during routine veterinary procedures.
Ethics oversight	Capture and collaring of wild camels within the Great Gobi Strictly Protected Area "A" was conducted within a cooperation agreement between the International Takhi Group and the Mongolian Ministry of Nature, Environment and Tourism signed on 15.02.2001 and renewed on 27.01.2011.

Note that full information on the approval of the study protocol must also be provided in the manuscript.
